# “Prescribing for the whole person”: A qualitative study exploring prescribing pharmacist views on type 2 diabetes management in New Zealand

**DOI:** 10.1186/s12913-023-09877-8

**Published:** 2023-10-04

**Authors:** Kimberley Norman, Shemana Cassim, Valentina Papa, Leanne Te-Karu, Penny Clark, Hilde Mullins, Lynne Chepulis

**Affiliations:** 1 Medical Research Centre, Te Huataki Waiora School of Health, University of Waikato, Private Bag 3216, Hamilton, New Zealand; 2 School of Primary and Allied Health Care, Monash University, Melbourne, Australia; 3 Te Kura Hinengaro Tangata School of Psychology, Massey University, Auckland, New Zealand; 4 Faculty of Medical and Health Sciences, University of Auckland, Auckland, New Zealand; 5 Northcare Medical Centre, Hamilton, New Zealand; 6 Department of Nursing, Te Huataki Waiora School of Health, University of Waikato, Hamilton, New Zealand

**Keywords:** Pharmacist prescriber, Inequity, Diabetes, Primary care

## Abstract

**Background:**

Pharmacist prescribers have comprehensive pharmacotherapy knowledge that can be useful for management of complex health conditions such as type 2 diabetes, yet the number of pharmacist prescribers working in New Zealand primary care is low.

**Aim:**

To explore the experiences of pharmacist prescribers in supporting type 2 diabetes management in New Zealand primary care.

**Methods:**

Qualitative research design using semi-structured interviews with six pharmacist prescribers working in NZ primary care. Thematic analysis guided this study and themes were finalised with the wider research team.

**Results:**

Three major themes were identified: team approach, health inequity and the role of a pharmacist prescriber. This study found that pharmacist prescribers may improve health equity by providing advanced pharmacotherapy knowledge within a wider primary care team to support complex patient needs and understanding the wider social determinants of health that impact effective diabetes management. Participants reportedly had more time to spend with patients (than GPs or nurses) and could also contribute to improving health outcomes by directly educating and empowering patients.

**Conclusion:**

The views of pharmacist prescribers have seldom been explored and this study suggests that their role may be under-utilised in primary care. In particular, pharmacist prescribers can provide specialist prescribing (and often mobile) care, and may contribute to improving health outcomes and reducing inequity when used as part of a multi-disciplinary team.

## Introduction

Pharmacotherapy is essential for optimising healthcare for many people, particularly those living with complex conditions like type 2 diabetes (T2D). Traditionally this has been the remit of General Practitioners (GPs) and nurses with specialised training (the latter within a limited scope of practice), with pharmacists historically restricted to dispensing prescribed medications or working as clinical advisory pharmacists (e.g. in hospital or primary care settings), where they influence pharmacotherapy including prescribing. However, more recently, there has been a move towards the development of pharmacist prescribers (PPs), practitioners who have undergone extensive speciality training in pharmacotherapy. PPs have been introduced into the healthcare workforce in countries such as the UK and Canada for some time [1]. This encompasses a range of pharmacist prescriber models and unique prescriber rights [2–4]. New Zealand (NZ) is considered to have an extensive training pathway to becoming a PP compared with other countries [1]. In NZ, the approval of legislation to allow PPs prescriptive authority has been relatively slow. Despite advocacy initiatives since 2004, the first cohort of PPs in NZ were not able to prescribe until mid-2013 [5]. Moreover, the pathway to becoming a PP in NZ is lengthy, taking a minimum of 8 years. PPs can be either highly specialised (e.g. in haematology or nephrology) or generalist, where prescribers can work in general practice across a range of health conditions, including T2D. Overall, the role of PPs is to facilitate access, optimal use, quality, safety, and efficacy of medicines under an umbrella of coordinated care [6].

Managing medications is essential for those with T2D, particularly as sub-optimal prescribing has been associated with reduced health outcomes [2]. T2D is a global epidemic affecting over 290,000 NZ adults, including a disproportionate number of Pacific and Indigenous Māori communities [7]. T2D, if left unchecked, can lead to further physical health complications, including eye damage, kidney failure, foot ulceration and heart disease [8]. While T2D is considered life-threatening, it is a treatable and sometimes preventable health issue [8] through effective management. One of the most effective strategies for managing T2D is optimum medication usage (coupled with dietary changes and weight loss engagement in some cases) [8, 9]. However, despite a range T2D medications being available in primary care, T2D rates [7] and the proportion of patients not meeting clinical targets [10] have continued to increase. Barriers to effective T2D management or optimal medicines management in this space may be contributing to inequitable health outcomes, especially for Māori [11, 12]. Local studies confirm what has been widely reported in NZ and internationally. One NZ study that suggests that more than half of all T2D patients may not meet glycaemic or cardiovascular risk targets [8], with others reporting reduced access to medications [13] and that reduced and inequitable prescribing of medications is involved [14, 15].

Research suggests that having a comprehensive and synergistic primary care team wrapped around T2D patients may be a valuable means by which health outcomes can be improved [16]. In particular, the involvement of PPs can lead to ongoing improvements in care for many patients, as they can provide valuable support for primary care. Involvement can include working alongside clinicians to manage diabetes patients, emphasising and managing the often complex pharmacotherapy needs of patients, thereby improving glycaemic control alongside other comorbidities, and improving health outcomes overall [17, 18]. Whilst PPs can offer invaluable knowledge for effective T2D management for patients in primary care, little is known about their experiences with managing T2D. In other countries, limited literature reports on the success of PPs for supporting T2D care [19–21], though this has not been evaluated in NZ despite PPs being integrated into healthcare for 10 years.

### Aim

To explore the PP experiences of managing patients with T2D, including the barriers and enablers that they faced working with these patients in NZ primary healthcare.

## Methods

### Data collection

This study was part of a larger T2D management in primary care study where clinicians (general practitioners, nurses and PPs) were recruited through general practice invitation to share their experiences about the impact of health system factors on the management of T2D in primary care. For this part of the study, purposeful sampling was initially used, with PPs identified across general practice and emailed to invite them to participate. A snowballing strategy was also employed where early participants were asked to pass on study information to anyone who might be interested to take part (with the latter then contacting us directly to take part). All potential participants interested in the study were given participant information sheets, and any concerns or questions were discussed before the participant agreed to participate. A suitable interview time and location was organised with the participant (all via zoom) and signed informed consent was obtained before the interviews commenced. Interviews were conducted by two researchers: KN (New Zealand European, female) and PB (Māori, male). The study was approved by the University of Waikato Health Research Ethics Committee reference HREC(Health)2022#19 and was completed in compliance with the Declaration of Helsinki.

### Participants

This study draws on the accounts of six PPs from the Auckland and Waikato regions of NZ. Due to the small number of PPs in these regions (and in NZ in general) we have not provided demographic information so as to maintain anonymity for these individuals. Participants comprised two groups: key informants (four interviews) and research advisors (two interviews). The rationale behind including observations from research advisors was that they have extensive knowledge, expertise and active experience in the area, thus providing context to supporting and strengthening the statements made by the key informants.

### Procedure

Interviews were semi-structured to ensure that, although guided by a set of questions, participants were able to take the conversation in any direction. Prior to commencing each interview, all participants were offered an opportunity to open the interaction in a culturally appropriate manner, such as through a prayer or karakia (this loosely means spiritual time of connection) []. Broad topics covered included participants’ experiences with T2D management with patients, health system factors that impact effective T2D prescribing and management, and any barriers or facilitators for prescribing for T2D in their practice. Interviews lasted between 30 and 80 min and were audio recorded. Participants were offered a $50 gift card as a thank you for their time.

### Analysis

All interview data was transcribed using transcription software (otter.ai) and checked manually for errors, accents, and colloquial terms. Preliminary analysis involved thematic analysis guided by the work of Braun and Clarke [22, 23] by three researchers (KN, VP, SC), independently and then together. Secondary analysis involved a meeting where the resulting broad themes and findings were discussed and debated with additional key research team members, comprising of five researchers: LC, KN, VP, PB, SC to ensure a reflexive and rigorous analysis process. This process involved comparison of themes, and robust discussion and re-analysing until a consensus was reached. This team of five included early, mid and late career researchers. Following this, the overarching themes were presented to the wider research team for their input.

## Results

Overall, participants in this study indicated many barriers in their roles of supporting effective T2D management, with narratives indicating that the complexities occurred at system, patient and prescriber levels. This study identified three over-arching themes to participant narratives; team approach, health inequity and the role of a PP.

### Team approach

Participants indicated that particularly for a complex lifestyle condition such as T2D, a team-based approach to care and T2D management was important. The PP role was positioned as a strong contributor to effective patient management in a primary care team approach, and one that had a number of advantages for people living with T2D, in addition to attending only clinical appointments with a GP or nurse. For example, PPs may have longer appointment times compared with GPs, enabling more time to address the complexities of T2D and educate patients. As participants describe:

*“So the advantage (of PPs) is that I have (extended) time to tell them (patients) why they’re taking the medicine” (Participant 2)*

*“The GP model of 15-minute appointments is just not conducive to managing a condition like T2D, we are clearly managing sugar levels, but also renal function, blood pressure, you know, the cardiovascular syndrome as well” (Participant 6)*

Having the ability to spend more time with patients and their ability to contribute advanced pharmacotherapy knowledge was reported to anecdotally be useful for other members of the primary care team. As highlighted by one participant:

*“(We received) feedback from the GPs and their colleagues about how useful it’s been having us (PPs). You need to have a multidisciplinary team to really enable effective prescribing as well. So it would be nice to have more integration. The beauty of having them (PP) in a (primary care) team is that you can be referred to people (in house). So that’s the strength of having pharmacists in the team, we can get asked questions all day, and also other people (doctors) will then see what I’ve been doing with other patients” (Participant 4)*

Working as a team, thereby enabled GPs, nurses and PPs to share information and advice, and support each other with their diverse and multidisciplinary skill sets. This approach also then allowed them to support patients as a team, which was highlighted to be important to improving patient health outcomes contributing to overall patient welfare. Importantly, in addition to the multidisciplinary clinical team, the patient also was considered to be integral to this ‘care team’. As highlighted by one participant:

*“I use ‘we’ all the time, so ‘we need to work together, our job together’. So it’s not putting the whole thing on the patient. It’s my role to support (patient). I really do pitch that as a partnership. It is really crucial to get a level of trust that will enable a good outcome” (Participant 3)*

Approaching their role as a partnership with the patient and the wider primary care team was highlighted to be useful for building trust in the therapeutic relationship, which in turn led to improved T2D management. This broad team approach where patients are also included through partnership also contributes to providing equitable care for people living with T2D, particularly when caring for Māori communities.

### Health inequity

PPs highlighted that an important aspect of their role was their ability to contribute to reducing patient barriers to healthcare by understanding the ‘whole person’, which was positioned as being central to engaging with Māori patients. As one participant expressed, creating time and space for culturally safe and respectful engagements was crucial if patient health was to be improved. Whilst time was brought up again as an enabling factor, it was the creation of an empowering relationship that was deemed essential:

*“I was very clear, when I started my rollout, that I wouldn’t be tied to a 15-minute appointment. You know, it takes 10 minutes to do whakawhanaungatanga (building relationships and connections) let alone, get into people feeling safe enough to pass over intimate health information” (Participant 6)*

Therein, participant accounts indicate that the flexibility they have with their appointment times allowed for them to engage in culturally respectful practice. This flexibility is something that GPs and nurses don’t often have with their appointment times.

The flexibility that PPs have not only with their appointment times, but also in how (and where) they engage with patients, also means that they can support patients who may be perceived as ‘hard to reach’ or ‘disengaged’. As a participant elaborates:

*“There was a patient that was really labelled as ‘disengaged’. So I met up with him, I bought him his coffee and spent (extra time) with him and his wife talking about it all through. He is now taking his medications. He wasn’t disengaged, we were disengaged with him” (Participant 2)*

Here, the unconventional approach that the PP had the ability to take when engaging with this patient was helpful and enabled the healthcare practitioner to really connect with the patient, and thereby revise the lens through which this patient was perceived. The reality of health inequity in NZ means that many Māori living with T2D do get perceived as being ‘disengaged’ and thereby do not get the support and care they need to manage their condition.

Further, some PPs stressed the accessibility of general practice was limited for people. According to participants:

*“We (health practitioners) are not open when patients are available” (Participant 2)*

*“(The system) is set up as, generally speaking, Monday to Friday, a nine to five sort of system. That’s not patient centred. And that presents barriers to lots and lots of people, especially if you’ve got financial difficulties. Or if you’re living far away from some of these rural areas, you’ve got distance to go to pick up the medicines once a month, some medicines, you have to pick up once a month, they can’t take three months at a time” (Participant 1)*

*“If you don’t have that ability to prescribe really well, then you’re never going to achieve equity” (Participant 4)*

Part of the issue with rigid appointment times and opening times is that it contributes to inaccessible healthcare for patients who work long hours (factory shift work, labour roles, truck drivers for example) and do not have flexibility in their roles to attend health appointments. Often many of these patients are part of high-risk populations, including high-deprivation, rural, and Māori. Having inaccessible healthcare for already high-risk groups further contributes to health inequity leading to poorer health outcomes.

Essentially, participants indicated that the PP is a complex role that cansupport and enhance how clinicians can care for people living with T2D, and how PPs have the ability in their role to meet the overall pharmacotherapy needs of patients.

### Role of a PP

PPs described many different facets to their role, noting their advanced knowledge and experience prescribing for complex comorbidities. A predominant aspect was their ability to treat the ‘whole-person’ in a holistic way, as highlighted by one participant:

*“The people I see with diabetes have usually got lots of other things as well (hypertension, kidney disease, gout), so having pharmacist prescribers who have a really in-depth knowledge of pharmacotherapy can prescribe for all of those conditions and make sure everything is appropriate. I am prescribing for the whole person. By addressing their needs you have an opportunity for them to start to trust you, and then have the opportunity to then look at the diabetes prescribing. That’s my starting point with most people, you know, getting to know them to be able to prescribe for them (effectively)” (Participant 4)*

Such a focus on the ‘whole person’ then allows participants to build trust with their clinician is a vital part of caring for people with an ongoing lifestyle condition such as T2D. Additionally, providing high-quality prescribing care required understanding their patient’s wider social determinants of health they live in. This is important as effective T2D management is not isolated to just managing blood glucose levels and medications, it is also impacted by a patients income, socioeconomic status, employment conditions and their social, familial and cultural responsibilities.

PPs also described that alongside the need to manage specialised pharmacotherapy, they also needed to deliver health information to patients. One participant describes how this delivery of information, and thereby supporting patients with their T2D management was a journey, that progressed step-by-step:

*“If we do a really good job at the beginning of explaining about the food and explaining about their what’s the basic pathophysiology of what’s going on for them, and the mechanisms of the medications or the reducing carbs, or the exercise and the conditions of the interventions that we’re recommending, then if we do that at the beginning, then as you go through, you build on that.” (Participant 2)*

It was widely recognised by these PPs that delivery of health information needed to be tailored to the patient:

*“If (diabetes information) hasn’t resonated with them, or they haven’t realised, (then) it hasn’t been told in a way that they could understand it” (Participant 4)*

One PP highlighted that tailoring health information delivery through diagrams or pictures was useful in their practice:

*“People respond to drawing. People of all abilities and all education from what I can understand. So I very rarely get anyone who says ‘stop there this is too much’. Or ‘I don’t understand anything you are talking about’” (Participant 3)*

Empowering patients with information and tools was positioned as part of the PP role and an effective strategy to assist patients with improving health outcomes, as expressed by one participant:

*“Honestly, people are very sharp, if they’re enabled or they’re empowered to be able to self-manage” (Participant 6)*

All participants positioned the role of PP as important for improving patient health and stressed the need for more PP roles to be funded in the future. As described by one participant:

*“Hopefully they’re trying to get funding because at the moment, there’s no specific national funding” (Participant 5)*

However, funding for more PPs was highlighted to only be one important factor for increasing the PP workforce. Participants stressed that any future development of PP roles needed to be actioned through appropriate training and qualification avenues, to ensure a high-quality workforce. As one participant detailed:

*“It’s about having a sustainable training pathway, so that you’ve got graduates or you’ve got pharmacists prescribers then who are appropriately trained. So, you can’t just do it (ad-hoc), or you can’t pull someone out from a dispensary and put them in a general practice. Because you’re not going to get any (or) see any benefits because they don’t-, they’re not clinically experienced, and or don’t have the necessary pharmacotherapy knowledge” (Participant 4)*

The role of a PP was considered to provide a valuable contribution to the primary care team. Their ability to provide comprehensive pharmacotherapy knowledge, time with patients, and understanding of how wider health factorscan affect patient T2D management and quality of life, enables them to assist with improving health outcomes, reducing inequity, and supporting clinicians and patients alike.

## Discussion

This study aimed to explore PPs’ experiences with T2D management and found three over-arching themes: team approach, health inequity, and the role of a PP. Overall, the role of a PP was positioned as a healthcare specialist who offers extensive pharmacotherapy knowledge and an invaluable skill repertoire to a primary care team. PPs have the ability to provide effective, patient-centred health management, whilst also offering significant support for clinicians experiencing high levels of workload and burnout (48% self-reported GP burnout rate in 2023 up from 31% 2020) [24, 25]. This study found that PPs can offer opportunities for breaking down barriers for patients accessing healthcare and clinicians providing this care, assisting with improving health outcomes, reducing health inequity, and alleviating some of the burden on primary care clinicians and practices [21].

The PPs in this study highlighted that they predominantly operate with a holistic or person-centred approach to patient healthcare, which has been identified previously as a vital component for improving health outcomes with patients [26, 27]. While this approach can be used by other clinicians in primary care, PPs have the ability to offer advanced pharmacotherapy knowledge coupled with an awareness of the wider social determinants of health that can impact patients’ T2D management. The PP has unique qualification pathways that develop specialised clinical skills [28] and was therefore likened to a specialist position that could offer significant expertise in a general practice context [28, 29]. In the UK, PPs are already long-established, providing strong support for improving patient health outcomes [30] as well as supporting an overburdened health system [31–33]. However, a PP is still considered an under-utilised role in NZ [34] and more research is needed to explore the patient and non-PP clinician views of the use of PPs in primary care teams.

The potential for PPs to contribute to improving health outcomes is promising, however there are only 46 practicing PPs in NZ serving a patient population of 5 million [35]. While the numbers for PPs have been slowly growing in recent years, PPs make up only 1.1% of all current registered pharmacists, indicating a strong need for more PP roles to be funded [35]. However, an increase in the PP workforce should occur carefully, where PPs need to be thoroughly trained and qualified and not pushed through the education system haphazardly to ‘fill a gap’ in the workforce. For instance, previous literature suggested clinicians were cautious over the ‘competence’ levels of PPs and that some development of a regulated qualification would be beneficial for patients and the primary healthcare team [36, 37]. However, these findings were based on predominantly international based literature from countries that do not have qualification pathways as extensive as NZ [37, 38]. In New Zealand, ‘competence’ must also include the ability to apply advanced pharmacotherapy knowledge in a culturally appropriate context, especially for indigenous patients and their family/whānau.

PPs appear to be effective and supportive members of the primary care team through their ability to provide high-quality care for patients and support clinicians with their patient workload. PPs are considered a feasible resource within the primary care team, which positively impacts the general practice environment, enables patients the ability to self-manage more effectively and contributes to alleviating the strain on the primary care workforce [17, 21, 38]. NZ is currently experiencing a health crisis with significant levels of staff shortages, limited time, funding and resources, and health reforms [39, 40]. Previous literature has indicated that there are mixed views about the role of a PP in a primary care team. One NZ study indicated some GPs were supportive of a PP providing care around medication review, but not as supportive in the PPs screening, monitoring or prescribing ability [41]. However, more recent studies have found that NZ GPs and nurses had strong levels of confidence in the pharmacist prescriber’s knowledge or ‘competence’ to manage complex patients with poorly controlled diabetes [17, 38] and highlighted the complementary potential that a PP has to the wider multidisciplinary team and patient care [17]. While patient perspectives are limited in NZ [34, 42] they do parallel studies in other countries [43] as well as the UK [18, 44, 45] which indicated that PPs were positively received by patients as useful members of the primary care team. In addition, PPs highlighted the importance of a patient-centred approach and the inclusion of the patient as part of the wider healthcare team, offering support for previous studies that recommend this strategy [26, 46].

PPs may also offer potential to assist with reducing the inequity experienced by many patients, including Māori when accessing T2D healthcare. Barriers to accessing healthcare for Māori are well known, including cost, the traditional Monday-Friday 9 − 5 health model, lack of time with clinicians, lack of transport options and a lack of culturally appropriate healthcare [10, 11, 47, 48]. Participants in this study expressed their potential to overcome some of these barriers with their flexible appointment times, ability to provide mobile care, minimal or no cost to patients and options for longer appointment times to include cultural customs and processes. Through this, PPs are able to optimise the benefits of medicines for patients and therefore contribute to decreasing medication related harm, which is reportedly common in primary care [49]. Further investigation (both quantitative and qualitative) into the potential extended benefits of having a PP in primary care is warranted, particularly for Māori who experience significant health inequity [13].

This study, whilst small, does have a number of strengths. Importantly, it provides valuable insight into the role that PPs can play in the support of primary care teams, including a deeper analysis of the day-to day lived and real-world experiences of these health professionals (something that has not been well described before). Further, we were able to conduct interviews with all the PPs working in the wider region (all from a range of backgrounds and workplace settings), and the views and findings presented in this study provide a strong indication that PPs may be highly beneficial for reducing health inequity and improving health outcomes in those living with T2D. However, we recognise that our study of six participants is small, and that as a result these findings may not be generalisable, either in New Zealand or overseas. As such, we suggest that further work is required to better understand the role of PPs for supporting patients with T2D and other chronic illness, including patient views of these services, and quantifiable measures of whether medication use and optimisation improves with PP involvement.

## Conclusion

PPs play an important role in supporting T2D management in primary care, including for Māori and those with complex medication needs. This study suggests that there is a need to more comprehensively explore the role of the PP workforce, including how they can be best be integrated into primary care to support management of T2D.

## Data Availability

The datasets generated and/or analyzed during the current study are not publicly available due the potential ability to identify participants but are available from the corresponding author on reasonable request.
